# Cyclic AMP dynamics in the pancreatic β-cell

**DOI:** 10.3109/03009734.2012.724732

**Published:** 2012-10-30

**Authors:** Anders Tengholm

**Affiliations:** Department of Medical Cell Biology, Uppsala University, Biomedical Centre, Box 571, SE-751 23 Uppsala, Sweden

**Keywords:** Epac2, insulin secretion, oscillations, protein kinase A

## Abstract

Insulin secretion from pancreatic β-cells is tightly regulated by glucose and other nutrients, hormones, and neural factors. The exocytosis of insulin granules is triggered by an elevation of the cytoplasmic Ca^2+^ concentration ([Ca^2+^]_i_) and is further amplified by cyclic AMP (cAMP). Cyclic AMP is formed primarily in response to glucoincretin hormones and other G_s_-coupled receptor agonists, but generation of the nucleotide is critical also for an optimal insulin secretory response to glucose. Nutrient and receptor stimuli trigger oscillations of the cAMP concentration in β-cells. The oscillations arise from variations in adenylyl cyclase-mediated cAMP production and phosphodiesterase-mediated degradation, processes controlled by factors like cell metabolism and [Ca^2+^]_i_. Protein kinase A and the guanine nucleotide exchange factor Epac2 mediate the actions of cAMP in β-cells and operate at multiple levels to promote exocytosis and pulsatile insulin secretion. The cAMP signaling system contains important targets for pharmacological improvement of insulin secretion in type 2 diabetes.

## Introduction

The pancreatic β-cells are adapted to respond to changes in the extracellular concentrations of glucose and other nutrients as well as hormones and neurotransmitters by releasing appropriate amounts of insulin to promote the uptake and storage of glucose in liver, muscle, and fat. Functional defects in the β-cells may lead to glucose intolerance and eventually clinically manifest diabetes mellitus. Insulin, like many other hormones, is released in pulses with a period of approximately 3–6 minutes (1-3). The pulses are important for the action of insulin on the targets, in particular the liver, probably by preventing down-regulation of the insulin receptors. The pulsatile pattern of insulin secretion arises from an endogenous rhythmicity of the individual pancreatic β-cell that involves several intracellular messengers, including ATP, Ca^2+^, phospholipid-derived messengers, and cyclic AMP (cAMP) (2,4,5).

There is consensus that glucose stimulates insulin secretion via its metabolism and ATP/ADP-dependent closure of ATP-sensitive K^+^ channels (K_ATP_ channels), which leads to membrane depolarization and opening of voltage-gated Ca^2+^ channels (6,7). The resulting increase of the cytoplasmic Ca^2+^ concentration ([Ca^2+^]_i_) triggers exocytosis of insulin secretory granules. Cyclic variations in cell metabolism and ATP/ADP ratio are thought to underlie the oscillations of [Ca^2+^]_i_ and pulsatile insulin secretion (2,4,8,9). Additional signals generated by glucose metabolism are important for a proper insulin secretory response by amplifying exocytosis at steps distal to the elevation of [Ca^2+^]_i_, but their identities have not been elucidated (7).

In addition to Ca^2+^, which is the primary triggering signal, cAMP is the most important regulator of exocytosis in β-cells. Cyclic AMP is a ubiquitous intracellular messenger involved in the regulation of a wide range of processes in many types of cells. The first indication that the nucleotide is involved in the β-cell secretory response came from the observation that cAMP-generating glucagon promotes insulin secretion (10), and other studies soon confirmed a link between cAMP and insulin release (11–14). It is now well established that cAMP promotes secretion at multiple levels, such as by increasing electrical activity and [Ca^2+^]_i_ signaling, by recruiting granules and by acting directly on the exocytosis machinery (previously reviewed in (15)). Cyclic AMP is also important for β-cell function by stimulating e.g. insulin synthesis, cell differentiation and proliferation, and by protecting the cells from apoptosis (reviewed in (16,17)).

**Figure 1.**
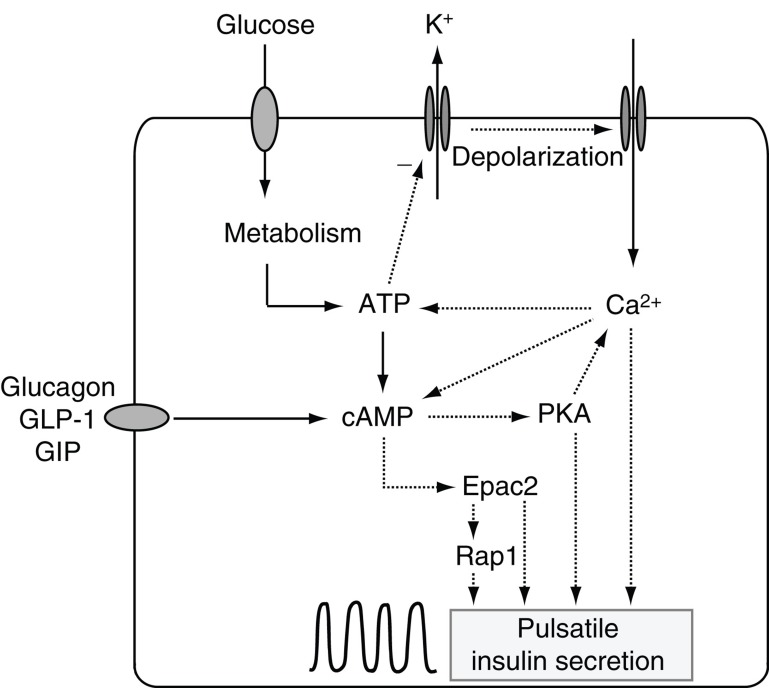
Cyclic AMP signaling in insulin secretion. Schematic drawing of a β-cell and the involvement of cAMP in insulin secretion stimulated by glucose and amplified by hormones. Glucose metabolism generates ATP, which inhibits ATP-sensitive K^+^ channels and causes voltage-dependent Ca^2+^ influx. Elevation of [Ca^2+^]_i_ triggers exocytotic release of insulin granules. ATP also promotes formation of cAMP, which amplifies secretion via Epac2 and protein kinase A (PKA). Activation of G_s_-coupled receptors by e.g. glucagon, GLP-1, or GIP leads to cAMP formation and enhancement of insulin release. Cyclic variations in metabolism, [Ca^2+^]_i_ and cAMP concentration caused by incompletely understood feedback circuits result in pulsatile insulin secretion.

The action of cAMP in β-cells is primarily linked to effects of certain hormones, in particular the glucoincretin hormones glucagon-like peptide-1 (GLP-1) and glucose-dependent insulinotropic polypeptide (GIP), which are released from intestinal L- and K-cells, respectively, to reduce postprandial glucose levels by enhancing insulin secretion. Cyclic AMP generation by receptor agonists has been reported to be required for normal glucose-responsiveness (18–20). However, as will be discussed in detail below, glucose also stimulates cAMP production in the absence of neuro-hormonal inputs. The effects of the nucleotide are mediated by protein kinase A (PKA) and exchange protein directly activated by cAMP (Epac), also known as cAMP-dependent guanine nucleotide exchange factor (Figure 1) (21). Despite the importance of cAMP in β-cell function, it is only recently that it has become possible to investigate the intracellular dynamics of the messenger. This review summarizes recent advances in our understanding of cAMP signaling dynamics in the context of insulin secretion.

## Cyclic AMP generation by adenylyl cyclases

Cyclic AMP is formed exclusively from ATP via adenylyl cyclases (ACs). The classical pathway for cAMP generation involves activation of transmembrane ACs by G_s_-coupled receptors. There are nine isoforms of transmembrane ACs with different regulatory properties (22). Most, if not all, of these are expressed in pancreatic islets and insulinoma cells (23–25). Early studies identified a close link between cAMP and Ca^2+^ (26–28), and particular attention has therefore been paid to AC isoforms regulated by this ion. The activities of AC1 and AC8 are stimulated by Ca^2+^ or Ca^2+^/calmodulin, and, despite relatively low expression, AC8 is functionally important by integrating G-protein and Ca^2+^ signals in β-cells (25). Recent studies also provided evidence that AC8 is required for GLP-1 generation of [Ca^2+^]_i_ signals (29). AC8 is preferentially found in raft-like domains of the plasma membrane where it interacts with the A-kinase anchoring protein AKAP79/150 and is regulated by store-operated Ca^2+^ entry (22,30–32). Although β-cells indeed exhibit store-operated Ca^2+^ influx, it is quantitatively minor compared to voltage-dependent Ca^2+^ entry (33), and its importance for AC regulation in β-cells is uncertain.

The more abundantly expressed AC5 and AC6 isoforms are inhibited by Ca^2+^ and by PKA-mediated phosphorylation. Little is known about their functional importance, but the regulatory properties indicate involvement in feedback inhibition of cAMP formation. Several ACs isoforms are regulated by protein kinase C, and there is evidence for a stimulatory effect of the kinase on AC activity in mouse islets (34–36). Recent data indicate that β-cell AC activity can be directly regulated by cell metabolism, probably via ATP (37). Apart from the nine transmembrane ACs there is a structurally distinct soluble AC (sAC) which at least is expressed in INS-1 cells (29,38) and which has been implicated in glucose-induced cAMP generation (38). In being regulated by Ca^2+^, HCO_3_^-^, and physiological concentrations of ATP (39–41), sAC seems well suited as a metabolic sensor, but functional evaluation of sAC is complicated by side-effects of the commonly used inhibitor KH7 (38,42). The importance of sAC for β-cell function therefore remains to be clarified.

## Cyclic AMP degradation by phosphodiesterases

The intracellular cAMP level is determined by a balance between cAMP production by ACs and degradation by cyclic nucleotide phosphodiesterases (PDEs). The PDEs constitute a large family of enzymes which catalyze the hydrolysis of cAMP and/or cGMP to 5′-AMP and 5′-GMP. There are 11 sub-families with >50 isoforms differing in structure, regulation, and substrate preferences (43). The role of PDEs in islets has previously been reviewed (44,45). The PDE1, PDE3, and PDE4 families are generally regarded as most important for cAMP regulation in islet cells. Pancreatic islets were early found to have Ca^2+^/calmodulin-sensitive PDE activity (46–48), and later studies have identified PDE1C as a prominent isoform (49–51). Pharmacological inhibition or genetic down-regulation of PDE1 thus enhances glucose-stimulated insulin secretion both from insulinoma cells and pancreatic islets (49,51).

PDE3 is a membrane-associated dual-specificity isoform degrading both cAMP and cGMP with kinetic properties that result in cGMP-inhibition of cAMP degradation. PDE3B is expressed in β-cells and is probably quantitatively the most important PDE in islets, constituting up to 70% of the total PDE activity in some studies (45,52). The enzyme is activated by glucose, insulin, and cAMP via changes in protein kinase A- and B-mediated phosphorylation (53). PDE3B is a major regulator of cAMP at sites important for insulin secretion. Overexpression of PDE3B in β-cells or insulinoma cells consequently reduces insulin secretion (54,55), whereas genetic down-regulation or pharmacological inhibition of the enzyme amplifies secretion (51,52,56,57), probably by regulating the most distal steps of granule fusion (58). Moreover, IGF-1-induced attenuation of insulin secretion is mediated by activation of PDE3B (56).

PDE4 is present in islets and insulin-secreting cells (51,52,59), but studies with inhibitors have yielded conflicting results. While the PDE4 family-selective inhibitor rolipram lacked effect on glucose-induced insulin secretion from islets (52,59), secretion was enhanced in both INS-1 cells and rat islets after selective pharmacological inhibition of the enzyme with roflumilast or L-826,141 and by siRNA-mediated knock-down of PDE4C (51).

Recent studies have also identified members of the PDE7, PDE8, PDE10, and PDE11 families in rodent and human islets and insulin-secreting cell lines (50,51,53). These PDE isoforms probably constitute a relatively small fraction of the total PDE activity in β-cells but may nevertheless play important functional roles. For example, pharmacological inhibition of PDE10A (60) and knock-down of PDE8B (50) potentiate insulin secretion from rat islets, and the latter isoform was recently implicated in cAMP oscillations and pulsatile insulin secretion from MIN6 cells (61).

## Cyclic AMP signaling triggered by neuro-hormonal stimuli

Several G_s_-coupled receptor agonists, including glucagon, GLP-1, GIP, pituitary adenylyl cyclase-activating polypeptide (PACAP), and ACTH, are known to enhance glucose-stimulated insulin secretion, effects which correlate with their ability to increase cAMP in β-cells (62–64). On the contrary, G_i_-coupled agonists like adrenaline, noradrenaline, somatostatin, galanin, ghrelin, and melatonin suppress insulin secretion, in part by reducing cAMP (65–68).

**Figure 2.**
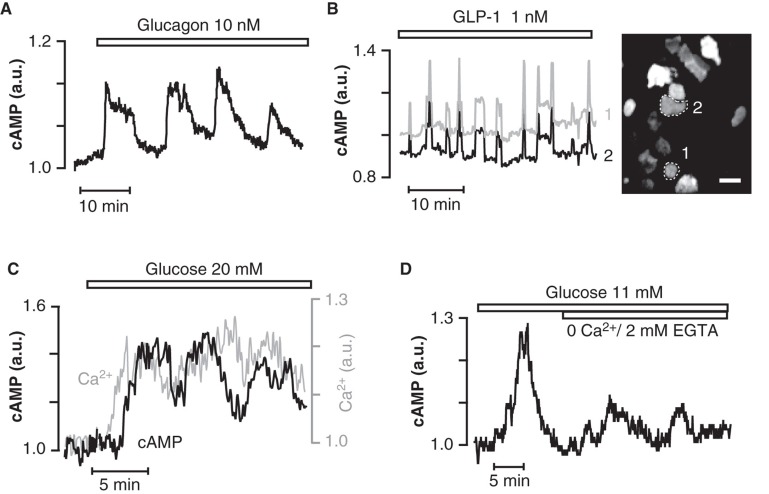
Cyclic AMP oscillations in hormone- and glucose-stimulated β-cells. Total internal reflection fluorescence (TIRF) microscopy recordings of the sub-membrane cAMP concentration in mouse β-cells within intact pancreatic islets. A, B: Cyclic AMP oscillations evoked by 10 nM glucagon and 1 nM GLP-1 in β-cells exposed to 3 mM glucose. Oscillations are synchronized among different β-cells within the islet as illustrated by graphs from the numbered cells in the TIRF image (B). C, D: Elevation of the glucose concentration from 3 to 11 or 20 mM evokes co-ordinated oscillations of cAMP and Ca^2+^ beneath the plasma membrane. The cAMP oscillations are amplified by Ca^2+^ but are maintained also when Ca^2+^ entry is prevented (D).

Measurements of single-cell cAMP dynamics beneath the plasma membrane have revealed that the β-cell cAMP response to glucagon and GLP-1 is oscillatory in both rat insulinoma cells (69) and primary mouse β-cells within intact islets (Figure 2) (42). Higher GLP-1 concentrations increase the time-average cAMP by prolonging the periods of cAMP elevation until the oscillations are replaced by stable elevation. The GLP-1-induced cAMP oscillations in insulinoma cells are synchronized with oscillations of [Ca^2+^]_i_ and abolished upon removal of the ion from the extracellular medium, consistent with a close connection between the two messengers (69). Such co-ordination of the triggering [Ca^2+^]_i_ and amplifying cAMP signals, which provides distinct stimulation of exocytosis, has been reproduced in modeling studies (70,71). However, elevated [Ca^2+^]_i_ is not necessary for the cAMP response to G_s_-coupled receptor agonists, since both glucagon and GLP-1 can trigger cAMP oscillations in mouse islets at sub-stimulatory glucose concentrations (42). The cAMP oscillations are synchronized among different β-cells within the islet, reinforcing the idea that β-cells are functionally coupled (72,73).

## Glucose-induced cAMP signaling

Glucose has long been recognized to increase the cAMP content of pancreatic β-cells (26,74–76), an effect regarded to be secondary to elevation of [Ca^2+^]_i_ (26–28). Since the magnitude was modest and cAMP alone was unable to stimulate secretion, the interest for cAMP as a messenger in glucose-stimulated insulin secretion declined. From experiments demonstrating that purified β-cells have lower cAMP content, glucose-induced cAMP formation, and insulin secretion than intact islets and that the cAMP content and insulin secretion are restored by addition of glucagon or glucagon-releasing α-cells, it was suggested that cAMP has a permissive role in insulin secretion and that the main effect of glucose is to amplify cAMP formation by glucagon (77,78).

Glucose has indeed been found to amplify hormone-induced elevations of cAMP, an effect attributed to the elevation of [Ca^2+^]_i_ (25). When it became possible to measure cAMP dynamics at the single-cell level it was shown that glucose also induces pronounced increases of cAMP in both clonal β-cells (37,79) and isolated primary mouse β-cells (37) devoid of paracrine influences. Landa et al. (79) observed that the glucose effect is strictly Ca^2+^-dependent and mimicked by depolarizing agents. However, when [Ca^2+^]_i_ oscillations are evoked by a combination of high glucose and tetraethylammonium the very pronounced peaks of [Ca^2+^]_i_ coincide with nadirs of cAMP, probably reflecting activation of Ca^2+^-sensitive PDEs (79,80). Measurements of cAMP in the sub-plasmamembrane space showed that glucose not only increases the cAMP levels, but that the cAMP concentration often oscillates in synchrony with [Ca^2+^]_i_ with a periodicity of 2–10 minutes (Figure 2) (37). In intact islets of Langerhans these oscillatory responses become synchronized among neighboring β-cells (42), and the co-ordinated cAMP and Ca^2+^ signals are critical for generating pulsatile insulin secretion. The glucose-induced cAMP elevation is amplified by Ca^2+^, but low-amplitude oscillations remain also after removal of extracellular Ca^2+^ or inhibition of voltage-dependent Ca^2+^ influx (37,42). Glucose also triggers cAMP elevation and often with oscillations under conditions when [Ca^2+^]_i_ is clamped by high K^+^ in the presence of the K_ATP_ channel-opener diazoxide, and a similar effect is observed with the mitochondrial substrate α-ketoisokaproic acid (37). Glucose-induced elevation of cAMP independent of Ca^2+^ has also been reported in β-cells from mice transgenically expressing a FRET-based cAMP indicator (81). Together, these data provide strong evidence that cell metabolism is a potent stimulator of cAMP production.

The mechanisms by which metabolism stimulates cAMP formation are unknown. Since cAMP is formed from ATP it seems likely that its concentration directly regulates AC activity. In support for this idea, lowering of sub-membrane ATP consumption by Na^+^/K^+^-ATPase inhibition was found to trigger cAMP elevation, and ATP stimulates cAMP formation in permeabilized MIN6 β-cells (37). A problem with the hypothesis is that the *in vitro*-K_m_ for ATP of the islet ACs is ∼0.3 mM (82), which is an order of magnitude below the ATP concentration believed to prevail in the cytoplasm. On the other hand, affinities *in vitro* may not properly reflect the ATP dependence in living cells. The soluble AC has a higher K_m_ for ATP (40), and experiments in INS-1 cells have indicated that glucose-induced cAMP production might be mediated by sAC (38). However, in both MIN6 and mouse β-cells the glucose-induced rise of cAMP is completely suppressed by a selective inhibitor of transmembrane ACs. The sAC inhibitor KH7 abolished both cAMP and [Ca^2+^]_i_ elevations, but this effect could be ascribed to an inhibitory effect on glucose oxidation unrelated to cAMP (42). Further work is required to clarify the mechanisms underlying the stimulation of cAMP production by cell metabolism. Available data obviously cannot exclude that ATP also may have indirect effects.

The cAMP oscillations are driven by variations in AC rather than PDE activity. Partial inhibition of PDEs with an intermediate concentration of IBMX thus induces cAMP oscillations in the presence of a sub-stimulatory glucose concentration, indicating that variations in the rate of cAMP production under basal conditions are balanced by degradation of PDEs (61). Variations in the rate of cAMP degradation do not seem to drive cAMP oscillations since they are prevented by an AC inhibitor. PDEs are obviously crucial for lowering cAMP levels during each oscillation cycle, but no isoform alone is responsible for this effect. Use of PDE-selective pharmacological inhibitors identified PDE3 and PDE1 as most important for shaping glucose-induced cAMP oscillations in clonal MIN6 and primary mouse β-cells. In addition, siRNA-mediated knock-down of the IBMX-insensitive PDE8B in MIN6 cells was found to perturb both cAMP oscillations and pulsatile insulin secretion (61).

Does cAMP account for the metabolic amplification of glucose-induced insulin secretion? The observations that glucose metabolism promotes cAMP accumulation (37,81) and that ATP can stimulate exocytosis at distal steps in a PKA-dependent fashion (83) are consistent with such an action of cAMP. On the other hand, with the observations that PKA is not involved in the amplifying pathway, that the correlation between cAMP and insulin secretion is sometimes poor, and that cAMP is ineffective in enhancing Ca^2+^-dependent secretion in the absence of glucose, it has been concluded that cAMP is not the main metabolic amplification signal (84–86). However, the studies have not taken into account that conventional measurements of average cAMP will underestimate the levels reached during the peaks of oscillations, in particular if the changes primarily occur in a specific sub-compartment. Moreover, these studies are typically based on insulin secretion evoked by high concentrations of K^+^, which may involve a different pool of granules than that induced by glucose (87). Further studies seem required to clarify if cAMP is or contributes to the metabolic amplifying signal or whether the two pathways are distinct and operate in parallel.

## Role of PKA in insulin secretion

PKA is a major effector of cAMP in β-cells, and the kinase is involved in mediating the stimulatory effects of the incretin hormones and other cAMP-elevating agents on insulin secretion. Many proteins have been identified as targets for PKA phosphorylation (reviewed in (15,88)). Anchoring of the kinase to specific sub-cellular localizations via A-kinase anchoring proteins is important for its actions on insulin secretion (89–93). PKA is highly dynamic, and cAMP oscillations have been found to be directly translated into oscillations of enzyme activity (80). The oscillations may contribute to keep signaling locally restricted. This idea is supported by the observation that brief elevations of cAMP do not provide sufficient time for the PKA catalytic subunits to diffuse through the nuclear pores and enter the nucleus, which requires prolonged cAMP elevations (69,80,94).

Cyclic AMP has long been known to promote β-cell electrical activity and Ca^2+^ signaling (95–97). The enhancement of [Ca^2+^]_i_ signals involves both voltage-dependent entry and intracellular mobilization (98–101) and can largely be explained by PKA phosphorylation of voltage-gated channels (102,103), K_ATP_ channels (18,104), and IP_3_ receptors (101,105). Effects of GLP-1 on intracellular Ca^2+^ stores have also been suggested to involve the Ca^2+^-mobilizing messengers cyclic ADP ribose and nicotinic acid adenine dinucleotide phosphate (106) and Ca^2+^-induced Ca^2+^ release via ryanodine receptors (107). These mechanisms were reported to involve both PKA and Epac.

Cyclic AMP also stimulates exocytosis by actions distal to the elevation of Ca^2+^ (102,108–110). PKA is involved in sensitizing the secretory machinery to Ca^2+^ (111). PKA also increases secretory vesicle mobility and accounts for replenishment of the readily releasable granule pool (112–114), in particular by increasing the number of granules which are highly sensitive to Ca^2+^ (115,116).

**Figure 3.**
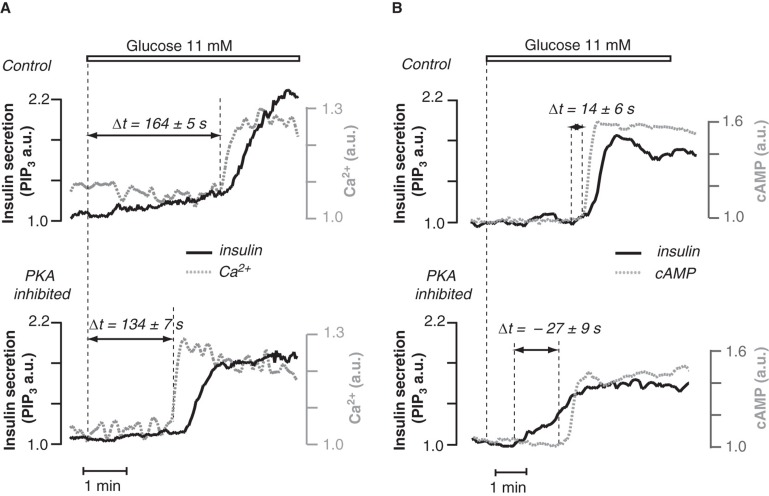
Temporal relationship between glucose-induced Ca^2+^ and cAMP signals and insulin secretion. A: TIRF microscopy recordings of sub-membrane Ca^2+^ concentration (dotted curves) and the insulin secretory response (solid curves) in MIN6 β-cells show that inhibition of PKA markedly shortens the delay between glucose stimulation and the initial Ca^2+^ elevation triggering secretion. B: Simultaneous recordings of cAMP (dotted curve) and insulin secretion (solid curve) showing that PKA inhibition shifts the timing such that secretion, which normally follows the amplifying cAMP signal, instead precedes the cAMP elevation.

**Figure 4.**
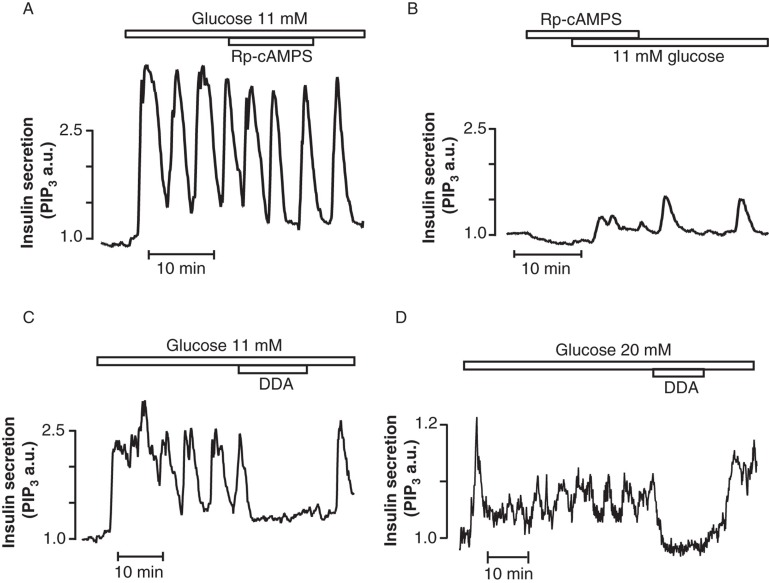
Cyclic AMP dependence of glucose-induced pulsatile insulin secretion. TIRF microscopy recordings of the insulin secretory response from individual MIN6 β-cells. A: The PKA inhibitor Rp-8-CPT-cAMPS (100 µM) barely affects pulsatile insulin secretion triggered by glucose. B: In contrast, if added prior to glucose stimulation, the inhibitor markedly suppresses the subsequent secretory response. C, D: Glucose-induced pulsatile insulin secretion critically depends on cAMP generation as 50 µM of the AC inhibitor dideoxyadenosine (DDA) inhibits secretion in both MIN6 (C), and primary mouse pancreatic β-cells (D).

Despite the undisputed importance of PKA in mediating cAMP signals on exocytosis, inhibitors of PKA have surprisingly small effects on glucose-stimulated insulin secretion from rat islets (117,118). The explanation may be that PKA is primarily important during initiation of insulin secretion, as shown by time-resolved measurements of insulin release from single β-cells using two-photon excitation imaging with polar tracers (119). A detailed analysis of cAMP action in glucose-stimulated MIN6 cells demonstrated that PKA controls the magnitude of the secretory response by affecting the co-ordination of Ca^2+^ and cAMP signals (Figure 3) (120). Although PKA promotes Ca^2+^ entry via voltage-dependent channels (102,121), inhibition of the kinase neither suppressed the glucose-induced [Ca^2+^]_i_ response nor the glucose-induced cAMP elevation. Instead, inhibition of PKA accelerated glucose-induced membrane depolarization, such that the resulting [Ca^2+^]_i_ elevation triggered exocytosis before the amplifying cAMP signal was manifested (120). One potential explanation for this effect is that both the channel-forming Kir6.2 and sulphonylurea receptor-1 (SUR1) subunits of the K_ATP_ channel under basal conditions are phosphorylated by PKA at sites that increase channel activity (122,123). The regulation of K_ATP_ channels by PKA is complex, and whether phosphorylation is activating or inactivating depends e.g. on the levels of intracellular ADP (124). This intricate regulation may perhaps explain the apparent paradox that GLP-1 stimulates β-cell depolarization by closing K_ATP_ channels via a PKA-dependent mechanism (18,125). PKA is thus required for establishing an initial insulin response to glucose stimulation, but PKA inhibitors lack effects on already established pulsatile insulin secretion (Figure 4) or on secretion triggered by Ca^2+^. The latter observations indicate that the cAMP-dependence of glucose-induced insulin secretion is mediated mainly by effectors other than PKA.

## Role of Epac in insulin secretion

While PKA was long regarded as the only cAMP effector in β-cells, it was evident that some cAMP effects on exocytosis are independent of the kinase (113). It was soon discovered that the PKA-independent effects of cAMP on exocytosis are mediated by Epac (126), a guanine nucleotide exchange factor for the Rap family of small GTPases (21). The role of Epac in insulin secretion has previously been reviewed (15,127–129). There are two Epac isoforms, Epac1 and Epac2, which are expressed in pancreatic islets (130–132), but it is mainly Epac2 that has been implicated in exocytosis. Of the three splice variants of Epac2, β-cells only express the full-length version (133).

Epac-specific cyclic nucleotide analogues have been found to amplify glucose-induced insulin secretion from INS-1 cells and from mouse and human islets (131,132,134). Although the specific activator does not activate PKA, its effect on human islets is prevented by inhibitors of PKA, indicating that PKA has a permissive role for insulin secretion in human islets. Capacitance measurements have demonstrated that Epac2 accounts for the rapid cAMP-dependent potentiation of exocytosis and that PKA has slower effects (113,114). Epac has also been reported to recruit granules to the plasma membrane (87,135) and together with PKA to stimulate granule-granule fusion events (135). The small GTPase Rap1 has been found to link activation of Epac2 to stimulation of insulin secretion, probably by stimulating the recruitment of secretory granules to the membrane, but the detailed mechanism of action has not been clarified (87). One possibility is that Rap1 activates Vav2 and Tiam (87), guanine nucleotide exchange factors for the small GTPases Cdc42 and Rac, which regulate insulin secretion via modulation of the actin cytoskeleton (136,137). Another alternative is that Rap1 stimulates mobilization of intracellular Ca^2+^ via activation of phospholipase C-ε (138). In support of the latter idea knock-out of phospholipase C-ε has been found to disrupt Epac-selective potentiation of insulin secretion (139). It has been suggested that cAMP stimulates intracellular Ca^2+^ mobilization primarily via Epac activation of ryanodine receptors (140,141), but this conclusion has been questioned (105). The study by Dyachok et al. (105) is instead consistent with the idea that cAMP-stimulated Ca^2+^ mobilization mainly occurs via a phospholipase C–IP_3_-mediated mechanism.

Epac was originally found to interact with the SUR1 subunit of the K_ATP_ channel (126). This interaction may result in modification of the ATP-sensitivity of the channel (142). Interestingly, the PKA-independent component of cAMP-stimulated secretion is absent in SUR1^-/-^ mice (114), suggesting that interaction between Epac and SUR1 is important for granule priming. Epac2 has also been found to bind to the Rab3-binding protein Rim2 (126,143,144), and this interaction is important for the stimulatory effect of incretin hormones on insulin secretion. Also the Ca^2+^-binding protein Piccolo, a neural active zone protein, is expressed in β-cells and interacts with Epac2 (145,146). In addition, interaction between Epac2 and the t-SNARE protein SNAP25 may be a prerequisite for the fast PKA-independent effects of cAMP on exocytosis (147). Whether these actions of Epac are mediated by Rap1 or not has not been determined.

**Figure 5.**
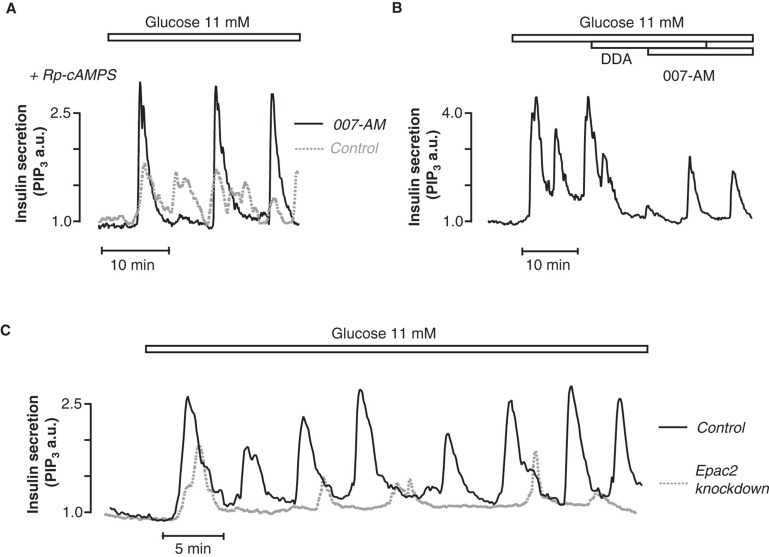
Involvement of Epac in glucose-induced pulsatile insulin secretion. TIRF microscopy recordings of the insulin secretory response from individual MIN6 β-cells. A, B: The Epac-selective cAMP analogue 8-pCPT-2’-O-Me-cAMP-AM (007-AM, 1 µM) restores the magnitude of insulin secretion initiated by glucose in the presence of the PKA inhibitor Rp-8-CPT-cAMPS (A), as well as that of established glucose-induced insulin pulses suppressed by AC inhibition with dideoxyadenosine (DDA) (B). C: Knock-down of Epac2 with siRNA reduces the magnitude of both initial and subsequent pulsatile insulin secretion in response to glucose.

Recent observations indicate that Epac2, in addition to mediating the amplification of insulin release by incretins and other cAMP-elevating agents, is involved in glucose generation of pulsatile insulin secretion (120). Thus, studies in MIN6 cells demonstrated that an Epac-selective cAMP analogue restored not only the initial glucose-induced insulin secretion suppressed by PKA inhibition, but also subsequent pulsatile secretion perturbed by adenylyl cyclase inhibition (Figure 5). Conversely, when the expression of Epac2 was knocked down by siRNA there was a marked reduction of both the initial and subsequent pulsatile insulin secretion. These findings contrast with results from Epac2 knock-out mouse islets where the glucose response is not significantly decreased despite markedly reduced cAMP amplification of glucose-induced insulin exocytosis (87,148). This discrepancy may reflect an inherent difference between MIN6 cells and mouse islets or that compensatory mechanisms are differently activated by the knock-down and knock-out strategies. However, there is no information on secretion dynamics from the knock-out islets, and secretion was either measured in static incubation experiments (148) or estimated by imaging of single exocytosis events (87). It has been reported that Epac regulates exocytosis of small synaptic-like vesicles rather than that of insulin-containing dense-core granules (149). However, this conclusion is supported neither by granule-imaging of knock-out mouse β-cells (87) nor by studies of the autocrine effects of insulin in MIN6 cells (120).

The sulphonylurea class of anti-diabetic drugs, which depolarize the β-cell by inhibiting K_ATP_ channel conductance after binding to the SUR1 subunit of the channel, was recently found to directly bind and activate Epac2 (148). This observation has gained support from another study, which even identified an Epac2 mutation that abolished the sulphonylurea interaction (150), whereas other studies have failed to demonstrate a direct interaction between Epac2 and sulphonylurea (151,152). A link between sulphonylureas and Epac2 activation is supported by suppression of the insulin secretory response to sulphonylureas in Epac2-knock-out mice (148). From the available data it is not clear whether the activation of Epac2 by sulphonylureas is direct or indirect, mediated for example by an increase of cAMP. Even if no overall elevation of cAMP was detected in the study by Zhang et al. (148), sulphonylureas may interact with PDEs (153,154) and thereby increase cAMP in local sub-compartments, which might be sufficient for Epac2 activation. Future studies will establish the nature of the link between sulphonylureas and Epac2.

## Cyclic AMP signaling in type 2 diabetes

Type 2 diabetes is characterized by loss of first phase and impaired second phase insulin release (155) with disappearance of the regular pulsatile secretory pattern (156). It is now broadly accepted that the disease develops as a result of β-cell dysfunction (157,158). It is not known whether cAMP generation is impaired in β-cells from patients with type 2 diabetes. However, several aberrations in diabetic subjects may be envisaged to affect β-cell cAMP handling, such as the reduced incretin effect and alterations of glucagon secretion (159,160). Single nucleotide polymorphisms that correlate with fasting blood glucose and type 2 diabetes have been identified in genes linked to cAMP signaling, including the GIP receptor, the α_2_ adrenergic receptor and AC5 (161–163). Alterations in β-cell cAMP signaling have been reported from several animal models of diabetes. Decreased glucose-induced cAMP generation and insulin secretion were thus found in diabetic Chinese hamsters (164), neonatal streptozotocin diabetic rats (165), and GK rats (166), and β-cell function was regained by treatment with cAMP-elevating agents (165–167). In some animal models of type 2 diabetes there are increased basal cAMP levels and exaggerated responses to AC activators, which may be linked to an increased expression of several AC isoforms (24,166,168–170) and decreased expression of PDEs (166). Altered cAMP handling has also been found after prolonged culture of β-cells in high glucose (29,171). INS-1E cells exposed to 20 mM glucose for 3 days showed reduced cAMP accumulation in response to forskolin and IBMX. Microarray analysis of gene expression showed several changes in the cAMP-signaling pathways, including a reduction of AC8, a finding confirmed also in rat and human islets (29).

Recently developed treatment strategies for type 2 diabetes are based on mechanisms that increase β-cell cAMP levels (reviewed in (172–176). The most successful approaches are based on activation of β-cell ACs via incretin hormones, but the rapid degradation of the hormones via dipeptidylpeptidase-4 (DPP4) is a problem. However, inhibitors of DPP4 increase the availability of endogenous circulating GLP-1 and GIP, and stable incretin hormone analogues as well as the GLP-1 mimetic exendin-4 have successfully been employed in diabetes treatment. The fatty acid receptor GPR119, a G_s_-coupled receptor predominantly expressed in islets, has also been identified as a promising drug target (174,177). Strategies based on inhibition of PDEs seem less promising due to low tissue specificity.

## Conclusions and future perspectives

Nearly half a century after the discovery of the link between cAMP and insulin secretion, cAMP signaling in β-cells is still a topic for intense research. Methodological advances in the past few years have provided novel insights into the spatio-temporal dynamics of the messenger and the regulation of its downstream effectors. It has become increasingly clear that the cAMP concentration often show complex spatio-temporal patterns that contribute to the versatility and specificity of the signaling. There are yet many unresolved questions. Future studies will clarify the detailed molecular organization of the local cAMP signaling circuits and the precise mechanisms by which PKA and Epac potentiate insulin secretion, particularly in human β-cells. Moreover, potential defects in cAMP handling of β-cells from diabetic human islet donors need to be explored. Cyclic AMP is important also for the release of other pancreatic islet hormones. Clarification of the intricate interplay between the different endocrine cell types in the islet is a prerequisite for fully understanding normal β-cell function and the pathophysiology of impaired hormone secretion in diabetes as well as for improving treatment strategies.

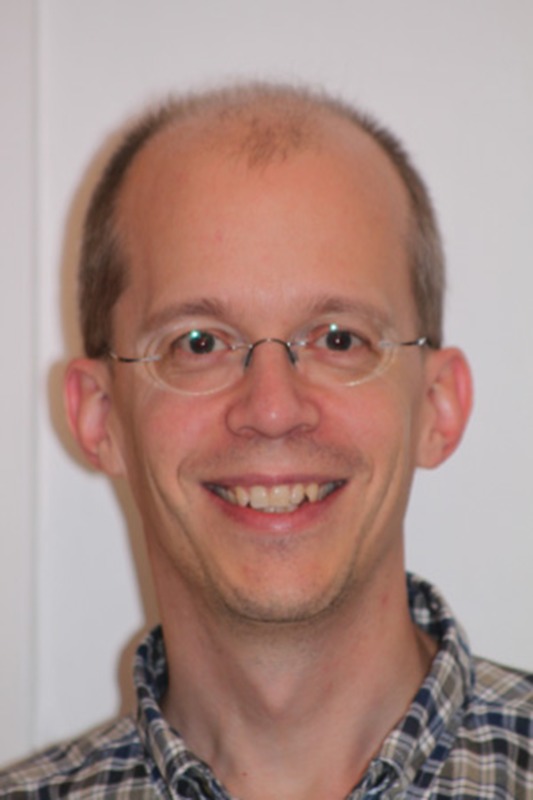
 **Winner of the Eric K. Fernström Award for young investigators 2011 at the Medical Faculty of Uppsala University.**
